# Learning leadership and feedback seeking behavior: Leadership that spurs feedback seeking

**DOI:** 10.3389/fpsyg.2022.890861

**Published:** 2022-07-22

**Authors:** Samantha Crans, Persiana Aksentieva, Simon Beausaert, Mien Segers

**Affiliations:** Department of Educational Research and Development, School of Business and Economics, Maastricht University, Maastricht, Netherlands

**Keywords:** leadership, learning leadership, feedback seeking, informal learning, quantitative method, qualitative method

## Abstract

Lifelong learning is crucial for professionals to continuously develop and update their knowledge and skills, and for organizations to create and sustain competitive advantage. In this regard, feedback seeking is a powerful vehicle to gain new knowledge and insights in one’s development and performance. The current research dives deeper in the concept of feedback seeking by investigating the act and use of feedback as well as multiple feedback seeking methods. Leadership as a contextual factor can affect employees’ feedback seeking behavior. As such, this study also explores the role of learning leadership for feedback seeking. Learning leadership supports, facilitates and encourages employees’ professional development. To address these aims, two independent studies were conducted. Study 1 was a quantitative, survey study that investigated the direct relationship between learning leadership and (the act and use of) feedback seeking. Study 2 was a qualitative, interview study that explored which concrete learning leadership behaviors were linked to different methods of feedback seeking. The findings confirmed the pivotal role of leaders in employees’ feedback seeking behavior and provided an overview of concrete learning leadership behaviors.

## Introduction

Organizations are faced with economic challenges, rapid technological advancements and globalization of markets. In this regard, investing in human capital through workplace learning ensures an adequately skilled workforce as well as sustainable competitive advantage for organizations (Jiang et al., 2012; Salas et al., 2012). Informal learning has been shown to be particularly effective for transferring acquired knowledge to practice (Sparr et al., 2017) and developing problem-solving skills through reflection of one’s work practices (Manuti et al., 2015). It is also linked to positive outcomes, such as employability (Froehlich et al., 2014a), innovative work behavior (Gerken et al., 2016), and job performance (Park and Choi, 2016). Indeed, as the review by Manuti et al. (2015) shows, during the past decades, many researchers plea for acknowledging the importance of informal learning as an essential component of workplace learning. Informal learning is defined as learning from experiences that are embedded in daily work activities and take a less formal form (Manuti et al., 2015). In other words, informal learning reflects the acquisition of knowledge and skills which is not as structured as formal learning, nor does it require predefined learning paths or learning objectives. It often occurs on as-needed basis and is initiated by the learner, often driven by a certain need to learn.

Informal learning takes place in various forms, such as an unplanned chat or planned discussion with colleagues, checking monthly company mails to employees with updates on organizational procedures, observing how an experienced colleague from a different department handles meetings, asking a colleague for help in tackling a challenge or asking a manager to give feedback on a draft report. These are examples of concrete informal learning activities which can be categorized in individual learning, social learning and learning from non-personal resources (Noe et al., 2013). Learning through social interactions with colleagues is of particular importance. Eraut (2004) stressed the potential benefits of social approaches to informal learning, such as seeking and receiving feedback, collaborating on team projects and observing colleagues and supervisors. Particularly feedback seeking, as a proactive social informal learning activity, has a pivotal role in learning (Crommelinck and Anseel, 2013). Seeking feedback enables the learner to receive and use information related to one’s performance or behavior. Feedback seeking has a meaningful impact on learning. It can contribute to deeper reflection of the self and one’s behavior as the feedback generated includes information that is oftentimes specifically aimed at improving one’s skills or performance (Crommelinck and Anseel, 2013), rather than merely solving a problem at hand or gaining information to fill a gap in knowledge.

To create conditions for employees to engage in feedback seeking behavior, the role of the leader has often been stressed. This is reflected in The LinkedIn Workplace Learning Report 2021 in which the manager is referred to as a “skill-building weapon” (LinkedIn Learning, 2021). According to the results of the survey published in this report, nearly half (49%) of L&D professionals are cooperating with managers to drive learner engagement and skill building. Throughout the years, feedback seeking research has also paid attention to the role of the leader. For example, Anseel et al. (2015) and Ashford et al. (2016) referred to specific leadership styles and the relationship between leaders and employees (i.e., leader–member exchange) when studying feedback seeking. Studies on leader–member exchange (LMX) highlight how high-quality relationships between leaders and their employees may increase feedback seeking behavior (Chun et al., 2014; Ashford et al., 2016). Well-established leadership styles such as transformational leadership and, to a lesser extent, authentic leadership also have been linked to feedback seeking (VandeWalle et al., 2000; Qian et al., 2012; Anseel et al., 2015). Although these former studies on leadership styles and feedback seeking are useful to understand why employees do or do not engage in feedback seeking they are often limited to well-established general leadership styles, such as transformational and authentic leadership (Qian et al., 2012, 2016; Anseel et al., 2015; Wang et al., 2016; Xie, 2019). However, when focusing on feedback seeking as a workplace learning strategy, literature specifically addressing leadership behaviors that positively or negatively influence learning at work offer a more relevant perspective in understanding how leaders can promote feedback seeking to support professional learning and development. In this respect, scholars use concepts such as learning leadership, learning-oriented leadership, leadership for learning, development-oriented leadership (Ellström and Ellström, 2018; Crans et al., 2021). Leaders who show learning leadership behaviors create conditions for professional learning, organize opportunities for employees to learn and motivate employees in their learning process (Wallo et al., 2021). A different stream of literature identified specific leader behaviors in the context of training. While research on leadership and transfer of training predominantly focused on leader behaviors that facilitate the transfer of what has been learned during training (i.e., formal learning) to the workplace, these leader behaviors might be relevant for informal learning as well. Govaerts and Dochy (2014) identified several types of supervisor support such as coaching what has been learned, supporting goal setting, giving feedback, having a positive attitude toward learning and tolerating mistakes.

Although a few scholars found that learning leadership is an important condition for engagement in informal learning, they address informal learning as a general concept without specifying the distinct informal learning behaviors such as help seeking or feedback seeking (Ellinger, 2005; Froehlich et al., 2014b). More specifically our understanding of the relationship between learning leadership and feedback seeking is limited. To address this gap, our study explores which learning leadership behaviors predict the engagement of employees in feedback seeking. In addition, prior research on feedback seeking mainly addressed the relationship between leadership and the frequency of feedback seeking (Crommelinck and Anseel, 2013). This stream of literature predominantly focuses on the act of feedback seeking and to a lesser extent on the actual use of feedback that has been sought. As Anseel et al. (2015) put forward in their meta-analytic review study, the act of feedback seeking is only one element in the process of feedback seeking. The current research will therefore also include the use of feedback seeking as well as feedback seeking methods.

In sum, the current study perceives leadership as an important situational factor and addresses the need to investigate concrete leader behaviors that encourage feedback seeking behavior (Anseel et al., 2015). Furthermore, it also responds to Bass and Riggio’s (2005) and Do and Mai’s (2020) call for more empirical research on leadership and informal learning, while not being limited by existing frameworks and theories on leadership and considering feedback seeking as a specific type of social informal learning. Our research aims firstly to study the direct relationship quantitatively between learning leadership and feedback seeking (i.e., act and use of feedback seeking; Study 1) and secondly to deepen our understanding of concrete learning leadership behaviors for learners to engage in different methods of feedback seeking behavior in a qualitative interview study (Study 2).

## Theoretical framework

Proactively seeking feedback from others has been argued as central in workplace learning (Eraut, 2000; Froehlich et al., 2014a; Gerken, 2016). For example, Schürmann and Beausaert (2016) illustrated in their study on drivers for informal learning that employees ranked feedback seeking and their interactions with and support from colleagues among the top three activities to learn informally. However, feedback seeking asks for an investment from the learner. It requires the learner to recognize the need for information on his or her performance and to subsequently engage in the proactive search for feedback (Eraut, 2000, 2004; Tynjälä, 2008). To support and facilitate learners to engage in feedback seeking, researchers have been addressing antecedents of feedback seeking behavior. In this regard, leadership has been argued to play a pivotal role (Ashford et al., 2016).

### Feedback seeking behavior

Feedback seeking is defined as the “conscious devotion of effort toward determining the correctness and adequacy of one’s behaviors for attaining valued goals” (Crommelinck and Anseel, 2013; p. 233). Inherent to this definition is the evaluative character of feedback seeking, referring to the need to search for information that can improve one’s skills, competences or performance. It is a process composed of one’s proactivity, interpretation, and their subsequent action (Ashford et al., 2003). However, prior studies predominantly investigated frequency of feedback seeking behavior and to lesser extent the use of feedback sought. Only if it is being used effectively, it contributes to employees’ performance (Renn and Fedor, 2001; Salas and Rosen, 2010; Van der Rijt et al., 2012).

Feedback seeking literature has focused on when, why, and how employees seek feedback (Ashford et al., 2003). As such, scholars mainly studied (1) feedback characteristics, (2) motives to engage in feedback seeking and (3) specific methods or strategies (Ashford et al., 2016).

#### Feedback characteristics

Historically, scholars interpreted feedback seeking characteristics as frequency and timing, source characteristics and topic of feedback sought. The frequency of feedback seeking refers to how often individuals engage in feedback seeking (Ashford et al., 2003). Timing of feedback seeking refers to the length of the time gap between an event (e.g., task performance or behavior) and the act of obtaining event-specific feedback (VandeWalle, 2003). This can be referred to as immediate or delayed feedback. The source characteristics may affect the costs and value that the feedback seeker attributes to feedback from a particular source. These refer to the source’s expertise and trustworthiness. A highly credible source may reflect a higher perceived value of the feedback and thus may increase the likelihood that feedback is sought from this source (Anseel et al., 2015). Finally, the topic of feedback refers to information about a process or outcome and might include failures or successes.

#### Motives of feedback seeking

Ashford et al. (2003) identified other elements of feedback seeking behavior, namely motives that drive employees to seek feedback in their environment: the instrumental, ego-based and image-based motives. The instrumental motive serves the purpose of regulating one’s behavior and attaining one’s goals (Ashford, 1986). The ego-based motive is driven by one’s intention to either defend or enhance their ego (Ashford et al., 2003). Professionals may refrain from actively seeking feedback if this carries the potential of wounding one’s ego. Lastly, the image-based motive refers to protecting or enhancing impressions that others hold of one. Based on these motives, one may attach potential costs, risks or benefits to seeking feedback.

#### Methods of feedback seeking

Finally, research started to focus on specific methods or strategies of feedback seeking. Ashford and Cummings (1983) identified two main strategies for seeking feedback, namely direct inquiry and monitoring. Most research to date has focused on these strategies (Anseel et al., 2015). Direct inquiry is the explicit and proactive verbal request for information. Monitoring, contrarily, is an indirect method that involves observing the source of feedback and the environment for cues indicative of one’s performance or behavior. Lately, De Luque et al. (2019) extended this research by adding five additional strategies: indirect inquiry (covertly asking questions related to performance), acting (spontaneously evaluating the current level of performance and how to retain it), backgrounding (giving information about the history of or path toward a task or current level of performance), forecasting (involving a future perspective in which one acknowledges the gaps in knowledge and deficiencies in performance and seek feedback on their plan of action), and opening (inviting the feedback source to give a candid opinion about a specific idea or focus of interaction by asking open questions. The current research focuses on explicit and direct feedback seeking methods, namely direct inquiry, monitoring, backgrounding, forecasting and opening.

Although feedback seeking behavior has been studied extensively, it is often assumed that the act of seeking feedback automatically results in useful feedback. This assumption remains untested, despite a call by Anseel et al. (2015) to further delineate the process of feedback seeking and to determine that feedback that has been sought, is also subsequently used. Furthermore, as Price et al. (2010) put forward, measures such as frequency or quantity of feedback only indicate that some conditions for useful feedback are present. It does not, however, indicate that this feedback is also effective. The current research addresses this concern by focusing on the act of seeking feedback and whether this feedback is also used.

Whether useful feedback is attained may also depend on the method or strategy of seeking feedback. For example, explicitly requesting feedback may elicit different information compared to merely observing the feedback source or environment which requires the feedback seeker to interpret these cues independently. Different methods might determine the usefulness of the feedback. In addition, scholars in the feedback seeking domain predominantly focused on two methods (i.e., monitoring and inquiry). To add to our understanding of feedback seeking, the current research also considers other active methods of seeking feedback.

To better understand which factors can influence feedback seeking behavior, prior research has devoted considerable attention to individual factors (VandeWalle et al., 2000; Anseel et al., 2015; Ashford et al., 2016) and situational factors, such as the relational context, supportive work environment, and leadership (Van der Rijt et al., 2012; Ashford et al., 2016).

### The role of leadership

#### Leadership that stimulates feedback seeking

When focusing on the role of leadership as a situational factor that influences feedback seeking behavior, prior research started investigating general leadership elements. VandeWalle et al. (2000) analyzed the influence of two leadership elements, namely leader consideration and leader initiation of structure. First, leader consideration reflects mutual trust, respect for employees’ ideas and consideration of their feelings (Fleishman and Peters, 1962). Second, leader initiation of structure refers to the extent to which a supervisor defines and structures work and job roles. Leaders who plan, communicate and monitor goals for work and performance, provide more structure for employees. These two elements highlight the role of a good relationship between leader and employee as well as providing a path with clear goals for employees to ask feedback about.

Moving forward, transformational leadership as a dominant leadership style received considerable attention in feedback seeking research. Indeed, some transformational leader behaviors are particularly relevant for feedback seeking. Individualized consideration, as an element of transformational leadership, refers to leaders who attend to their employees’ need and support them accordingly (Anseel et al., 2015). Leaders who demonstrate individualized consideration may simultaneously signal decreased costs associated with feedback seeking (VandeWalle et al., 2000). When employees feel their leader respects their individual needs, they might perceive the value of feedback seeking as a means to grow and might feel comfortable enough to engage in feedback seeking. Another aspect of transformational leadership, intellectual stimulation, is also linked to a decrease in costs. Leaders who provide intellectual stimulation challenge assumptions and stimulate employees to think deeply and find better ways to perform their work. In order to understand whether one is performing well, one can search for feedback on this process. Leaders who actually stimulate different ways of thinking and working, also signal the value of feedback seeking. A recent review by Anseel et al. (2015) showed that transformational leadership was related to higher frequency of feedback seeking. Similar findings were found by Wang et al. (2016) in their study on transformational leadership, trust and frequency of feedback seeking from supervisors. Transformational leadership was directly and positively related to frequency of feedback seeking, with trust also mediating this relationship.

In the past decade, however, scholars shifted their focus from traditional leadership styles to alternative conceptualizations of leadership (Ashford et al., 2016), such as the quality of the relationships between supervisors and employees (LMX; Chen et al., 2007; Chun et al., 2014) and supportive leaders who reduce perceived costs and increase the value of feedback seeking (Ashford et al., 2016). High-quality relationships (i.e., high-quality LMX) are characterized by mutual respect for each other’s capabilities, reciprocal trust, and the anticipation that work-related social exchanges will grow into a working partnership (Graen and Uhl-Bien, 1995). The working relationship between employee and supervisor will not have a mere functional character but also provides a foundation of trust and respect that, ultimately, facilitates feedback seeking behavior. Indeed, in a study by Chun et al. (2014) employees who experienced a high-quality relationship with their supervisor reported a decrease in perceived costs of feedback seeking and in turn increased the likelihood to engage in feedback seeking.

Building on prior research, one may assume that certain leadership behaviors promote or discourage feedback seeking. In their research Ashford et al. (2003, 2016) underline the role of leaders or supervisors in employees’ feedback seeking behavior. They argue that leadership style as well as relational context (e.g., supervisor–employee relationship) may decrease or increase the likelihood of feedback seeking in general as well as the methods or strategies used to seek feedback. Although there is a growing interest in the role of leadership, limited attention has been devoted to defining how exactly leadership affects feedback seeking behavior, what is understood as supportive leadership and which concrete leader behaviors may facilitate employees’ feedback seeking behavior.

#### Learning leadership

The concept of supportive leadership has been studied in many research areas related to professional learning, such as the domain of workplace (Marsick and Watkins, 2003), organizational learning (Xie, 2019) and the supervisor role in transfer of training (Govaerts and Dochy, 2014). Generally, these streams of literature see leadership as a way to support and stimulate employees’ performance and professional development in different contexts.

Learning leadership, also called learning-committed leadership (Ellinger, 2005) and learning-oriented leadership (Wallo et al., 2021) in workplace learning and organizational learning literature, are more geared toward leadership that facilitates workplace learning. Ellinger (2005) found that learning-oriented leadership had a positive influence on informal learning through peers as well as learning by doing. These leaders act as role models, while showing support and creating opportunities for learning (Ellinger, 2005). Research by Jeon and Kim (2012) demonstrate that top management encouraged informal learning through peers by emphasizing on a strategic level the importance of a skilled workforce and having a clear vision on Human Resource Development. Similarly, in a more recent study by Crans et al. (2021) learning leadership was found to be one of the prominent building blocks of a learning climate. Employees in this study perceived their direct supervisors as a pivotal factor in establishing an environment that fostered learning and development.

Another research domain that focuses on the role of the supervisor for learning is the transfer of training literature. Transfer of training refers to the extent to which employees effectively apply what they have learned during training in their job and work environment. Although training naturally covers formal learning, the transfer of training also includes elements of informal learning, such as applying new knowledge in the workplace, experimenting with new ideas and receiving feedback on this process. The role of supervisors is vital in the transfer of what has been learned during training to the workplace. As feedback seeking can be considered an informal learning activity and as the transfer of training literature has identified specific leader behaviors that might be relevant for informal learning, it is worthwhile to consider these behaviors too. Supervisors have a signaling function and an influential role in translating and implementing HR policies and practices. Employees may turn to supervisors for access to resources, trainings, and information. Supervisors, thus, can signal the value and importance of learning (Tracey et al., 2001) and provide means to participate in training or other learning activities (Govaerts et al., 2018).

These streams of literature continuously show the importance of leadership in professional development. Learning leadership focuses specifically on behaviors that stimulate employees’ development. Building on organizational learning, workplace learning and transfer of training literature, as well as research on well-established leadership styles such as transformational leadership, we define learning leadership as a leadership approach that facilitates, encourages, and supports employees in their professional development. Learning-committed leaders give feedback, stimulate critical thinking, encourage risk taking, facilitate social interactions, provide a psychologically safe environment and are a role model when it comes to continuous professional development.

#### Learning leadership as a leadership approach to facilitate feedback seeking behavior

Research on leadership behaviors that facilitate and support feedback seeking behavior among employees is sparse. Given that feedback seeking is also a social informal learning activity (Froehlich et al., 2014a; Gerken et al., 2016; Crans et al., 2021) and research in the domain of workplace learning, organizational learning and transfer of training more elaborately focuses on specific leader behaviors, it is presumable that learning leadership behaviors also positively affects feedback seeking behavior. As only few studies specifically focused on leadership in relation to feedback seeking, the current research draws from various streams of literature (i.e., feedback seeking, organizational learning, workplace learning, and transfer of training literature). Previous research found a positive relation between leadership and feedback seeking (e.g., Chen et al., 2007; Anseel et al., 2015; Wang et al., 2016). However, these studies are limited to traditional leadership styles. The current research takes a broader perspective, thereby aiming to define and conceptualize learning leadership. This perspective allows us to identify more leadership behaviors that are relevant for workplace learning and more specifically, and particularly relevant for the scope of this research, for feedback seeking.

In addition, when disentangling feedback seeking, learning leadership may be instrumental in the act of seeking feedback, the use of feedback that has been sought and the method through which feedback is acquired. Despite the broad array of research on feedback seeking behavior, it is often incorrectly assumed that the act of seeking feedback automatically results in feedback that is subsequently used. Several factors may determine whether feedback is effective and useful (Price et al., 2010). For example, the feedback seeker needs to understand and accept the feedback but should also be able to act on it. A leader who can facilitate this process may play an important role in the subsequent use of feedback. However, given our limited understanding of the role of learning leadership for feedback seeking, empirical examination of how learning leadership potentially affects the act and use of feedback seeking remains underdeveloped.

To further advance our knowledge of learning leadership and feedback seeking, the current research also considers feedback seeking methods. In the past, studies generally focused on only two feedback seeking methods. We broaden this scope by investigating how learning leadership behaviors relate to different methods of feedback seeking.

### Research aims and hypotheses

The general aim of the current research is to investigate whether a relationship between learning leadership and seeking feedback exists. In this light, two studies have been conducted. First, Study 1 quantitatively examines the proposed relationship and aims to generally explore to what extent learning leadership is related to feedback seeking as a proactive social informal learning activity. Furthermore, to further develop an understanding of feedback seeking behavior as well as the role of learning leadership, Study 1 investigates the relationship between learning leadership, and the act and use of feedback seeking. This results in the following hypotheses:

(1) Learning leadership is positively related to the act of seeking feedback.
(2) Learning leadership is positively related to the use of feedback that has been sought proactively.

Subsequently, based on our theorization and the results of the quantitative study, Study 2 qualitatively explores how feedback is sought (i.e., feedback seeking methods) and what the role of learning leadership is. In order to investigate the latter, learning leadership is conceptualized and measured in line with the different streams of leadership literature discussed above.

For Study 2 we address the following research questions:

(1) How do employees seek feedback?
(2) Which learning leadership behaviors co-occur with these feedback seeking methods?

## Materials and methods

### Study 1

#### Sample and procedure

A total of 228 employees contributed to this survey study. These participants worked at a Dutch food retailer (Sample 1; *N* = 132), a German firm operating in the medical technology industry (Sample 2; *N* = 74) and a German consulting firm (Sample 3; *N* = 22). The demographic information of all participants is shown in Table 1. The participants were approached by their supervisor, HR advisor or other contact person at the organization and were asked to participate in the study.

**TABLE 1:** Demographic information per sample.

	Gender		Age		Professional work experience		Tenure	
	Female in %	Male in %	*M*	*SD*	*M*	*SD*	*M*	*SD*
Sample 1	47.7	52.3	27.27	10.03	10.15	9.58	6.33	6.71
Sample 2	45.9	54.1	38.05	10.60	14.98	10.93	5.04	5.87
Sample 3	63.6	36.4	33.68	6.38	10.45	6.04	3.69	2.25
								

	**Level of formal education**							
	**Bachelor’s degree university of applied sciences in %**		**Bachelor’s degree university level in %**		**Master’s degree university level in %**		**Not specified in %**	

Sample 1	4.5		27.3		31.8		36.6	
Sample 2	40.9		22.7		5.3		31.1	
Sample 3	4.1		8.1		24.3		63.5	

#### Measures

##### Learning leadership

We used the validated ‘Learning Leadership’ subscale of the ‘A Climate for Learning’ questionnaire by Emonds et al. (2017) consisting of five items (e.g., ‘My manager looks for opportunities to learn new things’), rated on a five-point Likert scale (1 = strongly disagree; 5 = strongly agree). The scale was developed based on a literature review on learning climate. The internal consistency of this scale was satisfactory with a Cronbach’s alpha of 0.88.

##### Feedback seeking

Two scales of the ‘Proactive Social Informal Learning Scale’ by Crans and Beausaert (2020) were used to measure (1) the act of seeking feedback (four items, e.g., ‘I asked my colleagues to evaluate my work’) and (2) the use of feedback sought (three items, e.g., ‘I used the feedback that I received to work on my professional development’). All items were rated on a seven-point Likert scale, ranging from 1 = never to 7 = always or 1 = strongly disagree to 7 = strongly agree for the respective scales. The Cronbach’s alpha’s were 0.86 and 0.80, respectively.

##### Control variables

Based on the correlational analyses we selected ‘sample,’ ‘age,’ and ‘level of education’ as control variables for the hierarchical regression analyses.

#### Analyses

First, we applied correlational analyses to explore the relation between learning leadership and feedback seeking. Next, we performed hierarchical regression analyses to test the two hypotheses.

### Study 2

To capture a substantial amount of data and answer the “how” research question, a second qualitative study employing a Critical Incident Technique (CIT; Flanagan, 1954), was conducted. The CIT was preferred for this study because it simulates participants’ judgments on a particular experience which allows a detailed picture of why they acted and perceived the situation in a certain way. Furthermore, the description of an incident provides a comprehensive overview of the context in which the leadership behaviors and feedback seeking emerge together. These methods align with this work’s aim and allow exploring as many facets of the leadership behaviors as possible.

#### Sample and setting

To collect the data for this study 14 employees of different gender, age, nationality, and tenure all working at the headquarters of a Belgian scale-up company in the geospatial technology industry were interviewed. The employees had different job positions which enabled a heterogeneity in terms of their professional expertise and allowed gaining insights from various perspectives. For their participation in this study, the employees were contacted by the Talent Specialist of the company, and their contribution was voluntary. An inclusion criterion for taking part in the study was their participation in a feedback exchange.

In terms of demographics, the participants had an average age of 38 years (*M* = 38.36, *SD* = 9.05). Exactly half of the participants were female (50%) and half were male (50%). The tenure of the interviewed employees ranged from 6 months up to 25 years, which brought the average tenure to 8 years (*M* = 7.82, *SD* = 7.31).

#### Procedure

To collect the qualitative data, semi-structured interviews using the CIT were conducted (Flanagan, 1954). For their participation, the employees were contacted by the corporate Talent Specialist and required to think about three to four critical incidents before the interview. As a starting point in the interview, the respondents were asked to recall an experience that they could remember clearly about asking someone for feedback and receiving such. To be included in the analysis, these incidents had to be detailed and contain information on both feedback seeking and leadership behaviors that affected this behavior. Then, the participants were encouraged to provide more background information and reflect on the context guided by open-end and non-leading interview questions. Several follow-up questions were asked only if the participants did not provide sufficient information.

Nine interviews were conducted in a private face-to-face meeting at the company’s premises, and five interviews were held virtually by using video conferencing tool. The duration of the interviews lasted between 45 and 80 min. The audio of all interviews was recorded, and all participants gave consent to be recorded. The interviews were conducted by one data collector who neutrally asked the questions to prevent external influences and reactions to influence the outcome of the interviews (i.e., interviewer bias). The reliability of the method was ensured by using a systematized approach to data collection in that the interview sessions always started and ended in the same manner (i.e., controlled process using a protocol) and the questions asked were based on the interview guidelines.

#### Thematic analysis and coding process

After conducting all interviews, the data were transcribed verbatim. Subsequently, thematic analysis was applied to analyze the qualitative data (Braune and Clarke, 2006). Thematic analysis is a widely used analytic method that allows a theoretically flexible approach to analyzing qualitative data (Braune and Clarke, 2006). It is a method for identifying, analyzing and reporting of patterns (i.e., themes) within the data and it allows interpretations of various aspects (Boyatzis, 1998). These characteristics of the thematic analysis make it a suitable method for this study, as it allows discovering patterns and drawing qualitative conclusions on the interactions between learning leadership and feedback seeking behaviors.

The coding process followed the six phases (Braune and Clarke, 2006). First, all transcripts were read by the coders thoroughly. Next, the initial coding process was developed in which codes were generated deductively and inductively. During the process, overlapping codes were merged ensuring a clear differentiation between categories and themes. For example, the codes ‘asking probing questions’ and ‘suggesting new ways of working or dealing with a problem’ are behaviors that are categorized as ‘intellectual stimulation’ which is part of the general theme ‘providing developmental support.’ Finally, an extensive coding scheme was developed (see Tables 2, 3). The coding scheme was applied systematically to the data by using the scientific research software Atlas.ti. The data were segmented into units of meaning, and each was labeled with a code. The units of meaning which could not be assigned to any code because they contained ideas that were not covered by the pre-determined categories, were collected in a rest category. This category was, thereafter, analyzed and based on the content characteristics of the units, they were either assigned to already existing codes, or new codes were inductively created to cover the rest of the units.

**TABLE 2:** Coding scheme for learning leadership.

General theme	Subtheme	Behaviors	Example quote
Providing developmental support	Developmental Support	Coaching	“She said ‘we will have a discussion every morning, 5 min or 10 min on what you are doing that day and when you are struggling with priorities’.”
		Giving advice or feedback	“She is a manager who is very willing to receive and to give feedback”
	Intellectual stimulation	Re-examining assumptions and current ways of working	“So, we have the time to really look at things and to consider directions that might appear wrong at first, might still appear wrong after, but at least to go deeper into the first impression of some areas of the work we can do.”
		Suggesting new ways of working for dealing with a problem	“They [the managers] were giving suggestions for the next steps.”
		Asking probing questions	“She [my manager] said to me for example ‘for this task couldn’t you not delegate it to that person?”’
		Encouraging alternative or multiple viewpoints on an individual level*	“She already has years and years of experience and she also tries to make me aware of the fact that we give good services and that you can ask money for it and so I tried for myself to already from the beginning ask more of a budget [with which I feel comfortable].”
	Social interactions	Stimulating discussions and knowledge sharing	“You did not have to, but it was encouraged that everyone said something.”
		Encouraging alternative or multiple points of view	“And that is why we came together and also with the view ok, more people, more experiences, more points of view and probably they can bring across their way of working to the others.”
	Encouraging risk taking	Trying new or alternative ways	“Sometimes I get nervous because she says ‘simply take the telephone,’ but to me taking the telephone, it takes me also out of the things that I am working on and I need my bubble to work on things.”
Providing emotional support	Emotional support	Showing confidence, trust, recognition, understanding in employees’ learning and work	“I feel that she is listening to me.”
		Creating a psychologically safe environment	“I was kind of putting him in some words “above me,” but then he replied in a way to pull us at the same level by saying ‘I do not feel I am the right person for what I do’.”
		Informal reinforcement	“I know by reading through the lines that he appreciates me.”
		Caring for employees*	“Like the bosses, you feel they care about their employees, and he is one of them and so, yes, I feel supported”
		Good working relationship*	“I know him already from the beginning I worked here at Company X and I always had a good connection with him, so I also meet him outside work with a couple of ex-colleagues so if there is one person that I can talk to freely then it would be him.”
Providing practical support	Practical support	Providing time, resources, and venues for: identifying problems	“She said ‘we will have a discussion every morning, 5 min or 10 min on what you’re doing that day and during which time you are struggling with priorities’.”
		… organizational challenges	“They planned a meeting [to discuss these challenges within the organization| and they invited everyone.]”
		… reflection	“[My manager and I] have weekly sessions to discuss what I am doing and what the next steps are.”
		… improving performance	“I am just thinking for myself, ‘what do I think I can improve for this person?’.”
		… engaging in learning opportunities	“So, that is one of the first message that I always bring: let us work together to make that happen and it is only by exchanging that we can improve.”
Being a role model	Role model	Being a role model in work	“But what was really nice was that he opened up himself about his own job and he said, ‘I am not sure I am the right person for the new responsibility I have.’ […] And so that was pretty interesting to see. […] that he has feelings as well, and it was nice to go beyond the pure rational talking, it was making him very human.”
		Seeking feedback	“My manager is willing to give feedback and ask feedback.”
		Reflecting	“She said to me from the beginning that she knows she is not patient.”
		Being open to feedback	She said “if you have these things that are bothering you then come to me, just tell me and then we can talk about it.”
		Receiving feedback	“She listened to everything we discussed actually.”

*All behaviors marked with an asterisk are inductively created codes.*

**TABLE 3:** Coding scheme for feedback seeking.

General theme	Methods	Example quote
Feedback seeking methods	Direct inquiry	“I asked explicitly, ‘What do you think of this? And what is the feedback you want to give me?’.”
	Backgrounding	“And I just said, ‘Look, I am really struggling with where we are’ and I explained to him what my reasoning was: because I’ve tried this and I feel like it did not help you or then I noticed that after 2 weeks it was not implemented while in my opinion, it was only 3 days of work.”
	Forecasting	“In the end we defined some actions. So, first in a conversation just mentioning some ideas and some ways of tackling things or working on aspects and then, in the end, it was ok, and [subsequently], how can we now come to some specific actions to work on the next period.”
	Opening	“So, I just ask, ‘Have a look at my code’ and in some cases, I could say, and especially for this particular thing, I am not feeling very confident in that.”

To achieve reliability, the coding consistency was checked through an assessment of inter-rater reliability (IRR). An independent coder trained in conducting qualitative research coded 10% of the data. To measure the agreement between the coders, Cohen’s Kappa was calculated (Burla et al., 2008). This resulted in an IRR of κ = 0.743, which is indicative as substantial agreement (Landis and Koch, 1977).

To enrich the insights and give a nuanced, yet complete overview of the data, the frequencies of each theme were calculated, and the co-occurrences between the codes for feedback seeking and leadership behaviors were analyzed using Atlas.ti. The co-occurrences were explored from both a quantitative and qualitative perspective. The number of instances that two codes co-occurred in the data reveals how closely these codes are associated, but it is the exploration of the meanings of these associations that provides further qualitative insights.

## Results

### Study 1

Table 4 show the descriptive statistics and the results of the correlational analyses. The results show that learning leadership positively correlates to the act of feedback seeking and use of feedback. Furthermore, the demographic variables ‘age’ and ‘professional work experience’ are correlated to the use of feedback, but not to learning leadership nor the act of feedback seeking.

**TABLE 4:** Descriptives and correlational analyses.

Variable	*M*	*SD*	1	2	3	4	5	6	7	8	9
(1) Sample	2.42	0.80	X								
(2) Gender	0.51	0.50	–0.01	X							
(3) Age	31.39	11.10	0.19**	–0.10	X						
(4) Professional work experience	11.75	9.99	–0.02	–0.08	0.91**	X					
(5) Tenure	5.66	6.19	−0.29**	–0.03	0.53**	0.58**	X				
(6) Level of formal education	2.93	1.50	0.42**	0.03	0.30**	0.23**	–0.07	X			
(7) Learning leadership	3.45	0.87	–0.05	0.01	–0.02	0.00	–0.03	–0.12	X		
(8) Act of feedback seeking	3.62	1.20	−0.17**	–0.01	−0.26**	−0.21**	–0.08	−0.16*	0.18**	X	
(9) Use of feedback seeking	5.50	0.88	–0.02	–0.01	0.00	–0.00	–0.03	0.02	0.26**	0.33**	X

n = 228. *p < 0.05, **p < 0.01.

Hypothesis 1 predicted that learning leadership positively relates to the act of seeking feedback, while Hypothesis 2 predicted that learning leadership positively affects the use of feedback sought. The hierarchical regression analyses indeed confirm positive relationships (Table 5). The results also show that learning leadership is more strongly related to use of feedback compared to the act of feedback seeking.

**TABLE 5:** Regression results for the effects of learning leadership on feedback seeking.

	Act of feedback seeking	Act of feedback seeking	Use of feedback seeking	Use of feedback seeking
	Model 1	Model 2	Model 1	Model 2
Predictor	β	β	β	β
Sample	–0.11	–0.11	–0.03	–0.03
Age	−0.23*	−0.23*	0.00	0.00
Formal education	–0.05	–0.03	0.03	0.07
Learning leadership		0.17*		0.27**
*R* ^2^	0.09	0.11	0.00	0.07
Δ*R*^2^	0.09	0.03	0.00	0.07
F	7.00**	7.20**	0.10	4.29*

n = 228. *p < 0.01, **p < 0.001.

### Study 2

Table 6 reflects to what extent the interviewees engaged in different feedback seeking methods. Table 7 provides an overview of learning leadership behaviors that stimulate employees’ learning. The interplay between the leadership behaviors and feedback seeking was explored by analyzing the co-occurrences between learning leadership behaviors (i.e., developmental support, emotional support, practical support, and role modeling) and feedback seeking methods (i.e., direct inquiry, opening, backgrounding, and forecasting). The co-occurrences are shown in Table 8. The results of the co-occurrences will be discussed based on the most dominant co-occurrences.

**TABLE 6:** Frequencies and percentages of feedback seeking methods (*n*_1_ = 14 interviewees, *n*_2_ = 105 statements).

Feedback seeking methods	*n*_1_ (interviewees)	% (interviewees)	*n*_2_ (statements)	% (statements)
Direct inquiry	14	100%	58	55%
Opening	10	71%	22	21%
Backgrounding	10	71%	16	15%
Forecasting	6	43%	9	9%

*The codes are ordered according to the frequency with which codes mentioned.*

**TABLE 7:** Frequencies and percentages of learning leadership behaviors (*n*_1_ = 14 interviewees, *n*^2^ = 175 statements).

Learning leadership behaviors	*n*_1_ (interviewees)	% (interviewees)	*n*_2_ (statements)	% (statements)
**Providing developmental support**	14	100%	68	39%
Giving advice or feedback*	14		47	
Coaching	2		2	
Intellectual stimulation	9	64%	13	7%
*Encouraging alternative/multiple viewpoints on an individual level*	3		5	
*Asking probing questions*	3		3	
*Re-examining assumptions and current ways of working*	3		3	
*Suggesting new ways of working or dealing with a problem*	2		2	
Encouraging social interactions	3	21%	5	3%
*Stimulating discussions and knowledge sharing*	3		4	
*Encouraging alternative/multiple points of view*	1		1	
Encouraging risk taking	1	7%	1	0,6%
**Providing emotional support**	**13**	**93%**	**48**	**27%**
Having a good (working/personal) relationship*	10		20	
Showing confidence, trust, recognition, and understanding in employees’ learning and work*	8		18	
Caring for employees*	3		4	
Creating a psychologically safe environment	3		4	
Informal reinforcement	2		2	
**Being a role model**	**11**	**79%**	**33**	**19%**
In one’s work*	8		16	
In seeking feedback*	6		8	
In reflecting*	3		5	
In being open to feedback	2		3	
In receiving feedback	1		1	
**Providing practical support**	**8**	**57%**	**26**	**15%**
To reflect*	4		11	
To identify problems*	4		7	
To engage in learning opportunities*	5		5	
To improve performance	1		2	
In organizational challenges	1		1	

The codes are ordered according to the frequency with which codes mentioned. *All codes marked with an asterisk are leadership behaviors codes that co-occurred most frequently with codes for feedback seeking methods. The bold values correspond with the ‘General Themes’ in this Table 2 (i.e., providing developmental support, providing emotional support, providing practical support and being a role model). The behaviors following each bold marked theme are sub-behaviors related to that theme.

**TABLE 8:** Co-occurrences between learning leadership and feedback seeking methods.

	Developmental support	Emotional support	Practical support	Role modeling
Direct inquiry	17	15	7	8
Backgrounding	7	11	5	4
Forecasting	6	4	1	1
Opening	9	8	6	3
Total	**39**	**38**	**19**	**16**

#### Feedback seeking methods

All employees reported to directly inquire feedback. This code was also most frequently mentioned. This was followed by opening and backgrounding. Less than half of the participants reported to engage in forecasting. Not surprisingly, this code was also mentioned the least.

#### Developmental support

First, the category of providing developmental support was most frequently mentioned in relation to feedback seeking. Providing developmental support refers to concrete leadership behaviors that encourage professional development through coaching and giving advice or feedback, are intellectually stimulating, stimulate social interactions and encourage risk taking. In particular, the subcategory of giving advice or feedback was associated with the engagement of employees in feedback seeking. Employees particularly directly asked for feedback (i.e., direct inquiry).

*“I think he is a person who is very willing to receive and to give feedback and so I asked: ‘I would like a code review of my code, are you fine with that?”’ (Interviewee 2)*

This co-occurrence shows that if a leader is giving advice and feedback on a regular basis, it is more likely for their subordinates to feel comfortable enough to seek for feedback by more direct means, taking more risk.

On a similar note, giving advice or feedback was sought when an employee expresses not feeling competent or having low self-confidence (i.e., opening) and a competent feedback source is available:

*“So, a few days ago I was giving an introduction about the company and about our way of working to our new manager. I let him know throughout the conversation that I was a bit out of my depth because I have done onboarding for quite*

*a few newcomers, but always in sort of lower positions, job students or interns for example. I am quite comfortable giving a welcoming talk, but this is my manager. So, I said: ‘I am quite out of my depth here, so I hope I am doing ok.’ And in the end, he actually gave me some constructive feedback on it” (Interviewee 11)*

#### Emotional support

The second category which was mentioned often in relation to feedback seeking was emotional support. This category refers to leaders showing confidence, trust, recognition and understanding in employees’ learning and work, creating a psychologically safe environment, giving informal reinforcement, caring for employees, and having a good relationship with employees. When looking at the subcategories more specifically, a good working and personal relationship between the leader and employee was related to employees seeking feedback more often.

*“I would ask my manager about work in general. It is also because I think that she is willing to give feedback and that our professional relationship is okay, so I would ask her.” (Interviewee 2)*

In this example, the interviewee feels comfortable to use a direct method of seeking feedback (i.e., direct inquiry) because of their high-quality relationship. Due to those factors, employees might feel more confident and take more risk and thus use a direct inquiry approach.

Similarly, the interview data demonstrated that leaders showing confidence, trust, recognition and understanding in employees’ learning and work were more sought for feedback. Here, however, employees preferred to provide more information on what has happened and discuss past pathways (i.e., backgrounding).

*“I didn’t really ask him how I had to handle it, I just told him what happened and that things escalated. I trust him, I think he is a very good boss, I think his standards are very high, you know, he asks so much of you, but he appreciates the work that you do.” (Interviewee 14)*

In short, the findings demonstrate that showing confidence, trust, recognition and understanding creates a safe, trusting and understanding atmosphere. In this setting, employees might feel more inclined to provide background information and details on their feedback seeking attempt.

#### Practical support

The category practical support was mentioned less often in relation to feedback seeking behavior. Providing practical support includes leader behaviors such as providing time, resources, and venues for identifying problems, dealing with organizational challenges, reflection, improving performance and engaging in learning opportunities. In this category, leaders who provided time, resources and opportunities for reflection were linked most often to feedback seeking incidents. Also, leaders who provided resources for identifying existing problems, as well as learning opportunities played a role in employees engaging in feedback seeking behaviors. These three leadership behaviors (i.e., providing time, resources, and venues for reflection, identifying problems, and learning opportunities) were most often linked to direct inquiry and opening. The following quotes illustrate that the interviewee’s leader provides opportunities to reflect during weekly meetings and to identify problems together:

*“[My manager and I] have weekly meetings to discuss what I am doing and what the next steps are. I also just plan a meeting whenever I need to talk about specific problems that I cannot solve by myself, but also about the content of the work. But I also ask whether the content of my work is good or whether it needs to be improved.” (Interviewee 10)*

*“We also ask each other’s opinions, because sometimes a situation is not very clear, so then she will say ‘ok, you come over here and look at this problem with me,’ or the other way around.” (Interviewee 10)*

Sometimes a leader informs employees that they can just approach them and ask for feedback when needed. In this way a leader creates an opportunity for employees to improve and learn:

*“Before I had to prepare the PowerPoint presentation, we had a meeting together to discuss what should be in it and then he said: ‘if you want my feedback just come and ask me and then we will [discuss it together].’ [So] I went to him asking for feedback and to go over the presentation together. He gave me some very good ideas on how I could improve and what was missing in the presentation.” (Interviewee 13)*

#### Role modeling

Finally, a leader behaving as a role model was associated with fewest feedback seeking incidents. This category refers to leaders who are a role model with regard to their own work and development. These leaders engage in feedback seeking, reflection, are open to receiving feedback. Showing reflective behavior was linked to employees’ feedback seeking, followed by the ability and competence to execute work-related tasks and responsibilities (i.e., being a role model in one’s work), as well as the leader’s own feedback seeking behavior.

*“My manager is also very self-conscious about who she is and that makes it a very, very, a nice conversation in fact.” (Interviewee 5)*

*“We had a lot of people leaving the company last year. And I remember that during our weekly meeting we came to this issue that so many people left the company last year and then [my manager] asked: ‘what do you think is the reason [for them leaving]?’ I then gave feedback [on the matter] but also on his role in this situation.” (Interviewee 14)*

This finding demonstrates a reciprocity principle in which leaders who are good at what they do, engage in reflection and seek feedback from their subordinates themselves, play an active role in employees’ feedback seeking behavior in return. Due to their nature, all leadership behaviors in this category (i.e., showing reflective behavior, being good at their work and seeking feedback themselves) were associated with employees obtaining feedback in a direct manner (i.e., direct inquiry).

## Discussion

The current research aims to explore the role of learning leadership for feedback seeking as a social informal learning activity employing a quantitative and qualitative approach. The results of the survey study deepen our understanding of the extent to which learning leadership predicts the act of seeking feedback and the use of the feedback generated. The interview study results show in depth how learning leadership facilitate and stimulate feedback seeking behavior and which leadership behaviors play a prominent role.

### Discussion of the findings from Study 1

The aim of Study 1 was to quantitatively explore the relationship between learning leadership and the act of feedback seeking as well as the use of the feedback that has been sought. The findings of the quantitative, survey study confirmed that learning leadership is positively related to the act of seeking feedback and the use of feedback that has been generated by the learner. It adds to previous research that supervisors have a pivotal role in employees’ development. Many feedback seeking studies refer to the theory of a cost-value framework as underlying mechanism that determines whether employees engage in feedback seeking behavior. According to the cost-value framework, employees consider and assess the costs and values associated with aspects of feedback seeking behavior (Anseel et al., 2015). This research builds on review studies by Anseel et al. (2015) and Ashford et al. (2016) which put forward that leaders can decrease face-loss costs associated with feedback seeking behavior and can lead to higher frequency of feedback seeking. VandeWalle et al. (2000) aimed to develop an understanding of leader behaviors in relation to the frequency of feedback seeking behavior and concluded that two leader behaviors are related to a decrease in the perceived cost of seeking feedback. Leaders who value mutual trust, have respect for their employees’ views and ideas and are considerate of their feelings contribute to an environment and relationship in which an individual might feel more at ease to seek feedback. Our study not only confirms the importance of leader behaviors but also adds to this research by looking into the act of seeking feedback as well as the use of generated feedback. Furthermore, the role of learning leadership seems to be particularly important for the use of feedback that has been sought. This is even more relevant as prior research predominantly operationalized feedback seeking behavior in a general measure of frequency or act of seeking feedback and neglected the actual use of feedback (Crommelinck and Anseel, 2013; Anseel et al., 2015). The act of seeking feedback is not indicative of its effectivity, which partially depends on the response of the feedback seeker. How much value is attributed to the feedback and thus the likelihood that the feedback seeker also uses the acquired information, depends on whether the feedback is considered accurate, accepted, and processed by the feedback receiver. The degree to which feedback is used ultimately determines whether the employee will develop him or herself.

### Discussion of the findings from Study 2

The aim of the qualitative study was twofold. First, we explored how employees seek feedback (i.e., methods). Second, we identified which leader behaviors were relevant for stimulating and facilitating several active methods of feedback seeking. In order to do so, we also conceptualized learning leadership, bringing together different streams of literature (i.e., organizational learning, workplace learning, and transfer of training). We provide an overview of concrete learning leadership behaviors that are supportive of learning and identify four categories: developmental support, emotional support, practical support and being a role model. Leaders have critical role in providing support in different ways. First, developmental support refers to actions and behaviors that encourage and stimulate learning, such as giving advice or feedback, coaching, intellectual stimulation, stimulating social interactions and encouraging risk taking. Second, as identified in previous research a trusting relationship between leader and employee is essential (Chen et al., 2007). Providing emotional support includes having a good working or personal relationship, showing confidence and trust in employees’ learning and work performance, creating a psychologically safe environment, caring for employees and informal reinforcement. Another, yet essential way of facilitating learning is to provide practical support or otherwise understood as providing time and resources for identifying problems, organizational challenges, reflection, improving performance and engaging in learning opportunities. This is in line with leader behaviors mentioned by VandeWalle et al. (2000). Leaders who initiate, define and structure work encourage employees to seek feedback to clarify goals and monitor work processes. And finally, being a role model in one’s own work and learning journey signals exemplary behavior.

Turning to the role of these leader behaviors for the different feedback seeking methods, the findings show that all leader behaviors encourage employees to directly inquire feedback. We also see that some leader behaviors stimulate employees to use other feedback seeking methods. A good work relationship based on leaders who care for their employees and show trust and understanding for their work and development may encourage employees to give more background information when seeking feedback (i.e., backgrounding). These results are in line with research on LMX and relational context (e.g., Lee et al., 2007). As a leader one can foster these high-quality relationships and provide trust and understanding. In doing so, employees might feel more comfortable to engage in feedback seeking. Furthermore, employees who reported that their leaders provide advice and feedback (i.e., developmental support) and provide time and resources for employees to reflect, engage in learning opportunities and identify problems (i.e., practical support) were more inclined to invite the feedback source to give their candid opinion about their ideas and work and are less concerned with being viewed as incompetent or insecure (i.e., opening). Leaders can intellectually stimulate their employees while also considering their individual needs. Providing such developmental support encourages employees to reflect on their own growth and act accordingly.

The findings are in line with research on transformational leadership (Bass and Riggio, 2005), LMX (Lee et al., 2007; Chun et al., 2014), supervisor support during transfer of training (Govaerts and Dochy, 2014) and learning-committed leadership (Marsick and Watkins, 2003; Ellinger, 2005; Garvin et al., 2008; Emonds et al., 2017). The current study integrated different streams of literature to form a better understanding of the concept of learning leadership and, in turn, provide a more grounded account for the relationship between learning leadership and feedback seeking. This resulted in specific learning leadership behaviors, relevant not only for feedback seeking, but also for other informal learning activities.

### Limitations and directions for future research

As Anseel et al. (2015) argue, feedback seeking is a process during which various factors can be influential in different phases. As such, we suggest future research to also investigate the role of leaders in these different phases. More specifically, Anseel et al. (2015) propose a process model of feedback seeking in which several individual and situational factors are presumed to influence the different stages of feedback seeking. Starting with the feedback seeker’s prior attitude of and their motives to seek feedback, this may lead to a specific feedback seeking strategy or method. Subsequently, the response of the feedback provider determines the type of feedback that is being given (e.g., feedback on the process of outcome, valence, timing, and quality of feedback). The feedback seeker then determines if the feedback is accurate and valuable. If so, it needs to be processed on a cognitive level to ultimately be used effectively. Leadership, as a situational factor, may affect several stages. First, a leader can create an environment that encourages the act of feedback seeking among employees. As the qualitative inquiry showed, leaders may also have an influence on the feedback seeking method. Leaders can be the provider of feedback themselves, but they can also signal the importance of providing feedback by being a role model. The way the feedback provider and seeker respond to each other, may also depend on their relationship. For example, individuals who had a high-quality relationship with their supervisor sought more negative feedback from them (Chen et al., 2007), were more likely to seek feedback from them (Chun et al., 2014) and were more likely to engage in direct strategies (e.g., direct inquiry; Lee et al., 2007). For feedback to be effectively used and implemented, a leader may enable employees to put this new knowledge in practice by letting them experiment and implement this in their work.

The aim of the present research was to explore the relationship between learning leadership and feedback seeking. This micro focus on one learning activity allows for a deeper investigation of feedback seeking and its relation to learning leadership. However, it also disregards other informal learning activities. As leadership is considered one of the building blocks of a culture that fosters learning, it is particularly relevant to further dissect how leaders could behave in support of their employees’ development. We have aimed to integrate different streams of literature to form a better understanding of the concept of learning leadership. We implore future research to follow a similar approach and encourage future research to apply this conceptualization of learning leadership to other informal learning activities.

Furthermore, learning depends on the interaction between the learner and his or her environment. Learning climate is an example of a situational factor that determines to what extent learning occurs at the workplace (and thus feedback seeking). Learning leadership is an integral element of learning climate, perhaps even the most influential building block. It is, therefore, relevant to expand our knowledge on how to stimulate learning activities, such as feedback seeking, by also taking into account situational factors such as learning climate. On the level of the learner, individual characteristics such as motivation, goal orientation, age and experience may also play a role (VandeWalle et al., 2000; Anseel et al., 2015). This shows the complexity of any phenomenon at work and highlights the importance of building a comprehensive understanding of learning behavior and ways through which an organization can encourage and facilitate this. Future research may thus focus on the interplay between organizational and individual factors and their role in workplace learning.

Several methodological limitations should be addressed as well. First, the survey study was conducted in three organizations operating in different sectors, which resulted in varying sample sizes. This may hinder the comparability of these organizations. The interview study was conducted in a scale up company operating in an innovative field where there was a high need for communication and alignment. This may be different for larger organizations with institutionalized practices and structures in which this need for frequent communication and fine-tuning of performance is different. Furthermore, we were unable to report the response rate for Study 1. As such, response bias was not assessed. Future research may include larger samples taking into account company size, type and sector as well as more accurate sampling methods.

### Practical implications

The current findings can be translated into recommendations for organizations, HR professionals, leaders and employees. First, if organizations wish to adopt a climate in which learning behaviors such as feedback seeking are supported, their policies should devote specific attention to leadership development with learning leadership as a possible leadership approach. Leaders should be educated in the importance of their role and be equipped with tools and knowledge about how to support their employees in their development, bearing in mind the four categories of learning leadership behaviors (providing developmental, emotional and practical support as well as acting as a role model). Second, given that employees also have a great responsibility in their development and performance, they should be aware of why and how to seek feedback. Many feedback seeking methods, such as direct inquiry, opening, backgrounding and forecasting, can be applied, which may result in better performance and professional growth. Organizations can implement tools and structures that support feedback seeking, such as a learning management system or an online learning platform to educate employees in ways through which they can seek and use feedback.

### Conclusion

Our theorization and empirical findings confirm the importance of leader behaviors in feedback seeking. We add to different streams of literature by providing a conceptualization of learning leadership and defining concrete leader behaviors. More specifically, we expand extant literature on feedback seeking by specifically disentangling the role of leaders for feedback seeking, by highlighting the importance of actual use of feedback and by looking into more feedback seeking methods than generally has been done so far.

## Data availability statement

The raw data supporting the conclusions of this article will be made available by the authors, without undue reservation, to any qualified researcher.

## Ethics statement

Ethical review and approval were not required for the study on human participants in accordance with the local legislation and institutional requirements. The patients/participants provided their written informed consent to participate in this study.

## Author contributions

All authors have made a substantial and direct contribution to this work, contributed to manuscript revision, and approved it for publication.
